# Renal nutcracker syndrome presenting with varicocele: A case series

**DOI:** 10.1016/j.ijscr.2024.110393

**Published:** 2024-09-30

**Authors:** Hesam Mosavari, Milad Sarafi, Ali Hosseininasab, Nima Narimani, Mohsen Khaleghian, Ali Saberi

**Affiliations:** a Department of Surgery, Surgery Research Center, School of Medicine, Rasool-E Akram Hospital, Iran University of Medical Sciences, Tehran, Iran; b Hasheminejad Kidney Center, School of Medicine, Iran University of Medical Science, Tehran, Iran

**Keywords:** Case series, Gonadal vein transposition, Nutcracker syndrome, Saphenous vein bypass, Varicocele

## Abstract

**Introduction and importance:**

Nutcracker syndrome (NCS) is a rare condition characterized by left renal vein compression and presents with diverse clinical manifestations. This case series study aims to evaluate the clinical presentations and outcomes of patients with NCS and varicocele.

**Case presentation:**

We reviewed the cases of four male patients, aged 15 to 19 years, who presented with recurrent and grade 3 varicocele after varicocelectomy. All patients reported pain in the hypogastric, flank, or groin areas. Despite the absence of hematuria, NCS was diagnosed based on duplex ultrasound and contrast-enhanced computed tomography (CT) scans. Patients underwent open surgical interventions, including gonadal vein transposition or saphenous vein bypass.

**Clinical discussion:**

The absence of hematuria in these cases poses a diagnostic challenge, as current diagnostic criteria for NCS relies on its presence. Our findings underscore the need to consider NCS in patients with varicocele, even in the absence of hematuria. Surgical interventions were effective in resolving the symptoms. After NCS surgery, the varicocele in one patient resolved, and three patients underwent varicocelectomy successfully without recurrence throughout the recovery period.

**Conclusion:**

This case series highlights the diagnosis of NCS presenting with varicocele and local pain without hematuria, emphasizing the need for improved diagnostic and management approaches.

## Highlights

• Varicocele can be the initial and sole presenting sign of Nutcracker syndrome (NCS).
• Recurrent varicocele after varicocelectomy may warrant further imaging, to evaluate for NCS.
• Open surgical methods are effective in alleviating the symptoms and discomforts associated with NCS.

## 1 Introduction

Nutcracker syndrome (NCS) is a rare condition where the left renal vein (LRV) is compressed, causing reduced drainage into the inferior vena cava (IVC). This narrowing can be caused by external pressure from nearby structures, most commonly between the abdominal aorta and superior mesenteric artery (SMA) or vertebral column [1]. Although historically, NCS is known to present with hematuria and left flank or abdominal pain, varicocele is one the important manifestations that could be found in 15 % of patients [2]. Varicocele, in turn, can cause impaired fertility and scrotal pain and discomfort [3]. The link between NCS and varicocele stems from increased venous pressure in the LRV, causing venous hypertension. Decompression via an incompetent left gonadal vein redirects blood into pelvic or extra-pelvic veins, leading to varicocele. Thus, NCS can underlie varicocele, particularly in recurrent or atypical cases [2].

Diagnosis of NCS presenting with varicocele is based on excluding other possible causes. Moreover, the diagnosis of NCS is considered unlikely without evidence of hematuria [1]. This study aims to describe the clinical manifestations and outcomes of patients with NCS and varicocele who did not present with hematuria to emphasize the need for a better approach toward diagnosing and managing NCS.

## 2 Method

We conducted a retrospective case series study on four patients with varicocele and NCS referred to us at a tertiary academic medical center (Rasool-E Akram Hospital) between April 2022 and June 2024. This study followed the principles outlined in the Declaration of Helsinki and received approval from the institutional ethical review board. Written informed consent was obtained from all participants. The surgical case series was conducted per The Preferred Reporting of Case Series in Surgery (PROCESS) Guidelines 2023 [4].

### 2.1 Clinical presentation and diagnostic workup

The study included patients diagnosed with NCS upon further workup due to recurred left-sided varicocele after varicocelectomy. Data was collected from the medical records. All patients underwent a comprehensive diagnostic workup, including duplex ultrasound (DUS) and contrast-enhanced computed tomography (CT) scan. A urologist confirmed the diagnosis of varicocele, and based on physical examination, it was graded as follows: grade 1, palpated with Valsalva maneuver; grade 2, palpated without Valsalva maneuver; grade 3, easily visible [5]. The diagnostic criteria for NCS were based on previous studies [1,2,6] and are presented in Table 1.

**Table 1:** Imaging diagnostic criteria for NCS.

DUS findings
1. At least a 4-fold increase in distended to stenotic portions of LRV diameter ratio in an upright position
2. At least a 4-fold increase in distended to stenotic portions of LRV peak systolic flow velocity ratio in an upright position


Contrast-induced CT scan findings
1. Decreased aortomesenteric angle lower than 41°
2. >50 % reduction in the diameter of LRV at the site of compression
3. Beak sign (significant narrowing of the LRV at the aortomesenteric portion)
4. At least a 4.9-fold increase in hilar to aortomesenteric diameter of the LRV

LRV: Left Renal Vein, CT scan: Computed Tomography Scan.

### 2.2 Treatment

Disabling pain or discomfort, significant testicular hypotrophy (>20 %), progressive varicocele, or abnormal semen analysis were considered indications for surgery. Treatment options were discussed with each patient, and all patients underwent at least six months of conservative treatment. All patients underwent open surgery performed by a senior vascular surgeon with more than five years of experience.

All surgeries were performed under general anesthesia, patients were positioned supine, and a midline abdominal incision was made. In gonadal vein transposition surgery, the viscera were retracted to expose the retroperitoneum. The gonadal vein was mobilized by ligating small side branches and then transected distally in the pelvis. The vein was tunneled posterior to the inferior mesenteric vein, and intravenous heparin was administered. A partially occlusive clamp was placed on the IVC, and a tension-free end-to-side anastomosis of the gonadal vein to the IVC was performed with running sutures. Post-clamp release, Doppler assessment confirmed venous flow through the transposed gonadal vein and the new anastomosis.

In saphenous vein bypass surgery, the left renal vein and its surrounding structures were carefully exposed. The great saphenous vein was harvested from the patient's leg. Intravenous heparin was administered. The saphenous vein was tunneled from its harvest site to the retroperitoneal space. A partially occlusive clamp was placed on the renal vein, and a tension-free end-to-side anastomosis of the saphenous vein to the renal vein upstream of the compression site was performed with running sutures. The other end of the saphenous vein was anastomosed to the IVC. Doppler assessment confirmed venous flow through the bypass graft and the anastomosis.

### 2.3 Post-operative follow-up

After surgery, all patients were prescribed anticoagulation therapy (20 mg of Rivaroxaban daily) for one month and lifelong aspirin therapy (81 mg daily). Patients were followed up for a minimum of six months via outpatient visits. The primary goals of follow-up were to monitor for symptom resolution, including the absence of pain and recurrence of varicocele, as well as to assess for any potential complications. In addition, fertility outcomes were evaluated through semen analysis. The outcomes measured during these visits focused on pain relief, varicocele recurrence, and any adverse events related to the surgical intervention or anticoagulation therapy.

## 3 Results

We evaluated four patients with left-sided varicocele and NCS (Table 2). All patients were male with a mean (range) age of 17.25 (15–19) years and a mean (range) body mass index (BMI) of 17.65 (16.9–18.5) kg/m^2^. Patients had no underlying health conditions, were not taking any medication, and did not report any history of smoking. One patient (patient #3) had a history of inguinal hernia repair 11 years ago.

**Table 2:** Patients' demographic and clinical information.

Patient	Age (yr)	BMI (kg/m^2^)	conservative treatment period	Pain Location	Semen analysis		signs on CTS	NCS type	Surgery
					baseline	Post-operation			
1	16	16.9	1 yr	Suprapubic & groin	Azo	Oligo	Beak sign	Ant	GVT
2	19	17.2	6 mo	Flank	Azo	NL	>50 % reduction	Post	SVB
3	19	18.5	6 mo	Suprapubic & groin	Oligo	NL	Beak sign	Ant	GVT
4	15	18	6 mo	Flank	Oligo	NL	Beak sign	Ant	GVT

BMI: Body Mass Index; Azo: Azoospermia; Oligo: Oligospermia; NL: Normal; CTS: Computed Tomography Scan; NCS: Nutcracker Syndrome; Ant: Anterior; Post: Posterior; GVT: Gonadal Vein Transposition; SVB: Saphenous Vein Bypass.

All patients' initial complaints included pain in the hypogastric, flank, or groin area and grade 3 varicocele. No patient reported a history of gross hematuria, and their urine analysis was negative for microscopic hematuria and proteinuria at the time of their first visit to the urologist.

**Fig. 1:**
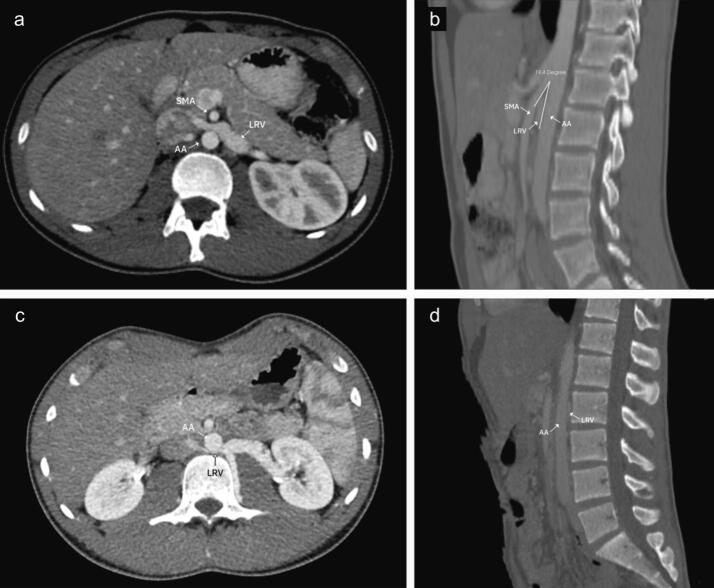
Preoperative Computed Tomography scan image of patients with anterior and posterior nutcracker syndrome (NCS). a) Anterior NCS - Axial view showing compression of the left renal vein (LRV) between the abdominal aorta (AA) and the superior mesenteric artery (SMA) (beak sign). b) Anterior NCS - Sagittal view showing the angle between SMA and AA is measured at approximately 19.4°. c) Posterior NCS - Axial view showing compression of LRV between AA and vertebrae body. d) Posterior NCS – Sagittal view.

Following their diagnosis of varicocele, all patients underwent subinguinal varicocelectomy. However, the varicocele recurred post-surgery, prompting further workup. This additional evaluation with DUS and Contrast-enhanced CT scan led to the diagnosis of NCS in these patients (Fig. 1, Table 2).

**Fig. 2:**
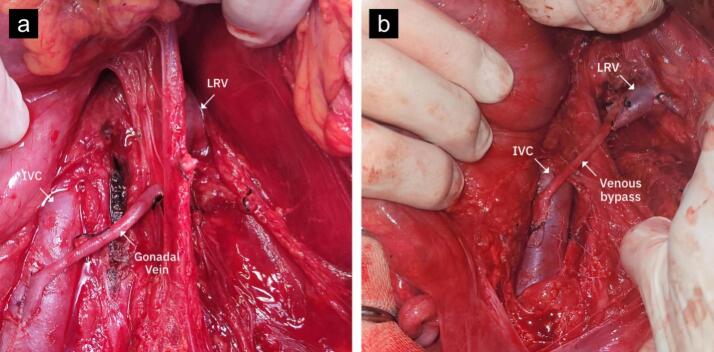
Intraoperative photographs. a) Gonadal vein transposition in a patient with anterior nutcracker syndrome. b) Saphenous vein bypass in a patient with posterior nutcracker syndrome. LRV: Left Renal Vein; IVC: Inferior Vena Cava; SMA: Superior Mesenteric Artery.

Three patients suffered from anterior NCS, and one had posterior NCS. Patients underwent open surgery with either gonadal vein transposition (patient #1, 3, 4) or saphenous vein bypass (patient #2). Patient #2 was initially planned to undergo gonadal vein transposition, but due to difficult access to the gonadal vein, the saphenous vein bypass procedure was utilized (Fig. 2).

The recovery period was uneventful for all patients, and they were discharged in good condition to resume daily life. Patients visited the outpatient clinic for follow-up visits at one week, three months, and six months. No post-operative complications, were reported during the follow-up period.

After a 3-month follow-up period following NCS surgery, patient #3 experienced complete resolution of the varicocele. However, in three other patients, the recurrent varicocele and symptoms persisted. These patients subsequently underwent successful local re-ligation, with no recurrence or complications observed.

## 4 Discussion

Clinical manifestations of NCS are widely variable, and there is a lack of unified diagnostic criteria, making the diagnosis challenging and still mainly remains a diagnosis of exclusion [1,2]. The most common clinical presentations of NCS are flank, abdominal, or pelvic pain (71.5 %), hematuria (69.5 %), and varicocele (15.8 %) [2]. In this case series, all of our patients initially presented with flank or groin pain and grade 3 varicocele without the classical presentation of hematuria.

Diagnostic approaches have long dismissed NCS without hematuria [1,7]. However, a recently proposed diagnostic algorithm recommended imaging studies for NCS if any primary symptoms - hematuria, abdominal, pelvic, or flank pain, congestion, or varicosities - are present, and more common conditions have been ruled out [2]. Initially, our patients underwent varicocelectomy without any further work-up for NCS. They experienced varicocele recurrence under three months and a temporary pain alleviation that only lasted for about one month. It has been reported that varicocele patients with NCS are at higher risk of recurrence after varicocelectomy [8].

A correlation between a lower BMI and NCS has been described in studies [1,8,9]. Similarly, our patients had a low BMI, with a mean BMI of 17.65 kg/m^2^. Conservative treatment, which includes weight gain, medical therapy, and lifestyle modification, has been proposed to be effective in the majority of patients under 18 years of age [7], as it may lead to the widening of the acute aortomesenteric angle, thereby alleviating compression of the LRV [1]. However, despite our conservative treatment plan focusing on weight gain, difficulties in achieving significant weight gain, along with persistent pain and progressive varicocele in our patients (during the 6–12 months of conservative management), ultimately led them to opt for surgical intervention.

NCS tends to present most commonly during adolescence and young adulthood, though it can develop at any age, from childhood through later life. The condition often peaks in individuals during the second and third decades due to rapid anatomical changes during puberty, such as the narrowing of the aortomesenteric angle, which increases the likelihood of left renal vein compression. In this case series, all patients were between 15 and 19 years old, which is consistent with the age group where NCS is typically diagnosed [7].

Surgical intervention for NCS, including left renal vein transposition, gonadal vein transposition, and saphenous vein bypass, has been shown to alleviate symptoms in most patients effectively. Studies have reported significant symptom resolution, such as cases of flank pain and varicocele, following surgical correction [[10], [11], [12]]. In a case series, 91 % of patients experienced relief from flank pain, and 82 % saw improvement in abdominal pain after surgery [10].

Between open surgical procedures for NCS, left renal vein transposition is the most frequently reported; however, left gonadal vein transposition has gained preference due to several advantages. These include technical feasibility, applicability to all types of NCS, avoidance of renal vein clamping, and the need for only a single anastomosis. Additionally, saphenous vein bypass offers an alternative for patients with both anterior and posterior NCS when neither the LRV nor the gonadal vein can be transposed [1]. Similar to the gonadal vein transposition, this method requires only brief clamping of the renal vein without clamping the renal artery [12].

Further research is warranted to better understand the prevalence, risk factors, and optimal management strategies for NCS. Developing consensus guidelines based on robust evidence will help standardize the diagnosis and treatment of this rare condition, leading to improved patient outcomes.

## 5 Conclusion

NCS presenting with varicocele can be a diagnostic challenge due to its variable and atypical clinical manifestations. This case series highlights the importance of considering NCS as a potential cause of varicocele, even without classical signs such as hematuria. Open surgical interventions, including gonadal vein transposition and saphenous vein bypass, were effective in relieving symptoms and improving patient outcomes.

## Ethical approval

This study was carried out following the principles outlined in the Declaration of Helsinki and received approval from the institutional ethical review board on the ethics code [IR.IUMS.REC.1402.1174].

## Funding

Authors received no funding.

## Author contribution

Milad Sarafi: Conceptualization, Methodology, Supervision

Hesam Mosavari: Conceptualization, Methodology, Writing - Original Draft, Writing - Review & Editing

Ali Hosseininasab: Investigation, Formal analysis, Data Curation, Writing - Original Draft

Nima Narimani: Investigation, Writing - Review & Editing

Mohsen Khaleghian: Conceptualization, Methodology, Project administration

Ali Saberi: Writing - Original Draft.

## Guarantor

Milad Sarafi.

## Registration of research studies

N/A

## Consent

Written informed consent was obtained from all individual participants or their parents. Additionally, written informed consent was obtained from all individual participants for publishing their data.

## Conflict of interest

Authors declare they have no conflict of interest.

## Data Availability

The data that support the findings of this study are available from the corresponding author upon reasonable request.
